# Development of the PROMIS-based Research Assessment and Clinical Tool-Fatigue (ReACT-F)

**DOI:** 10.1007/s00520-018-4614-2

**Published:** 2019-01-11

**Authors:** Kristin A. Dickinson, Debra Lynch Kelly, Jin-Shei Lai, Leorey N. Saligan

**Affiliations:** 1 College of Nursing, University of Nebraska Medical Center, 985330 Nebraska Medical Center, Omaha, NE 68198-5330 USA; 2 National Institute of Nursing Research, National Institutes of Health, Bethesda, MD USA; 3 College of Nursing, University of Florida, Gainesville, FL USA; 4 Feinberg School of Medicine, Northwestern University, Chicago, IL USA

**Keywords:** Fatigue, Cancer, Psychometrics, Questionnaire design

## Abstract

**Purpose:**

Evidence has shown that cancer-related fatigue (CRF) may be a treatment-limiting symptom and often impairs health-related quality of life. Accurate assessment of the multidimensional nature of CRF could help drive interventions to mitigate this debilitating symptom. Currently, there are no clinical tools to effectively and efficiently assess the multidimensionality of CRF. The purpose of this paper is to introduce a CRF-specific short form that can assess the multidimensional nature of CRF for use in the clinical setting.

**Methods:**

The CRF-specific short form was developed using the 95-item PROMIS® fatigue bank. Bi-factor analysis was used to evaluate dimensionality of the alternative model using fatigue for the general factor and physical, cognitive, affective, global, and motivational for the local factors. After unidimensionality was confirmed (loading factor > 0.3), one item from each local factor was selected using discrimination power for inclusion in the CRF-specific short form.

**Results:**

The Research Assessment and Clinical Tool-Fatigue (ReACT-F) was created from the 95-item PROMIS fatigue bank using established item parameters. The ReACT-F assesses five common dimensions of CRF as well as perceived burden of the fatigue dimensions.

**Conclusions:**

The ReACT-F is a CRF-specific self-report short form that addresses the need for a brief, clinically useful tool to quickly assess the multidimensional nature of CRF. We anticipate that the ReACT-F can be completed in the clinical setting in approximately 3 minutes, providing clinicians with meaningful data to drive personalized interventions. Further validation of the ReACT-F is highly encouraged.

## Introduction

Cancer-related fatigue (CRF) is a highly prevalent, complex, multidimensional symptom that can greatly impair the health-related quality of life of cancer patients [1, 2]. Clinical guidelines have adopted a single-item, 0 to 10 numeric rating scale to initially screen for CRF due to its easily administered nature [3–6]. Several guidelines recommend a more comprehensive evaluation when patients rate their fatigue ≥ 4 (i.e., moderate fatigue) using a 0–10 numerical rating scale. This evaluation includes a focused history, assessment of treatable contributing factors (anemia, nutrition deficits, pathologic/physiologic abnormalities, etc.), and concurrent symptoms (pain, depression, sleep disturbance, etc.) and conditions (cardiac, renal, pulmonary, etc.) [3–6]. Moreover, inclusion of a measure of the multidimensional nature of CRF would be advantageous to understand the full fatigue experience of cancer patients.

The fatigue experienced by cancer patients is often reported to be multidimensional in nature including physical, emotional, and cognitive dimensions, although the exact terminology for each dimension can vary (i.e., affective, motivational, behavioral, functional, etc.) [7, 8]. Therefore, when conducting an in-depth evaluation of CRF, clinicians should consider the multidimensional nature of CRF to fully capture the CRF experience and optimize management. Consistent with the Precision Medicine Initiative of the National Institutes of Health (NIH), understanding the specific dimension of CRF that most affects the patient can help guide the clinician to develop a more tailored and personalized management strategy.

Though comprehensive multidimensional fatigue assessments are available (e.g., revised Piper Fatigue Scale, Multidimensional Fatigue Inventory, and the Fatigue Questionnaire), most of them were developed using classical test theory resulting in measures that may not be best suited for a clinical environment [9]. Applications from the PROMIS® (Patient-Reported Outcomes Measurement Information System, http://www.healthmeasures.net) fatigue item bank are expected to overcome these limitations [10]. As part of the NIH’s roadmap project, the PROMIS® was developed to offer a set of person-centered measures to evaluate symptoms of individuals with or without chronic conditions [11]. One of these measures is the PROMIS instrument that assesses fatigue and the impact of fatigue on daily living [10]. The PROMIS fatigue bank consists of 95 items generated from a comprehensive literature review, focus groups, and individual interviews which were then calibrated using item response theory (IRT) models [10, 12], allowing for brief-yet-precise fatigue estimation via tailored, individualized computer adaptive test (CAT), or short forms with fixed numbers of items. For the latter, multiple short forms can be created to meet users’ needs and scores from these short forms are comparable as long as scores are generated using item parameters established in the original calibrated item banks. Yet precision levels may vary as demonstrated in Lai et al. (2011) in which three short forms were developed targeting patients with mild fatigue, severe fatigue, and for fatigue across the whole severity continuum. Several short forms derived from the PROMIS fatigue item bank are available [13–15], yet none of them target fatigue content areas that are important to cancer patients. Therefore, to fill this void, a content-specific CRF short-form was developed that can be used in the clinical setting.

## Methods

### Fatigue dimensions

To determine the fatigue dimensions of interest, current multidimensional fatigue assessments were reviewed (Table 1) [16–21]. The most commonly assessed fatigue dimension was physical (20/20) followed by cognitive (16/20), affective (7/20), global (6/20), and motivational (5/20). Thus, these five dimensions were selected moving forward. The *physical* dimension of CRF was conceptualized as fatigue related to energy level. The *cognitive* dimension of CRF was conceptualized as fatigue related to thought processes, memory, and executive function. The *affective* dimension of CRF was conceptualized as fatigue related to emotions or feelings. The *global* dimension of CRF was conceptualized as encompassing the subjective experience of fatigue. Lastly, the *motivational* dimension of CRF was conceptualized as fatigue related to actions that maintain a meaningful or purposeful existence.

**Table 1:** Multidimensional instruments for assessing fatigue domains

		Fatigue dimension					
	General Global Perception Fatigue Subjective Experience	Physical Activity Somatic Motor Energy Sensory Vigor	Cognitive Mental Concentration	Psychosocial	Motivation Behavioral Task Avoidance	Affective Emotional	Other
Bristol Rheumatoid Arthritis Fatigue Multidimensional Questionnaire		x	x			x	Living with Fatigue
Cancer Fatigue Scale		x	x			x	
Checklist of Individual Strength	x	x	x		x		
FACES	x	x					Consciousness Energized Sleepiness
Fatigue Impact Scale		x	x	x			
Modified Fatigue Impact Scale		x	x	x			
Fatigue Scale for Motor and Cognitive Functions		x	x				
Fatigue Questionnaire*		x	x				
Multidimensional Fatigue Inventory	x	x (2)	x		x		
Multidimensional Fatigue Symptom Inventory (MFSI)	x	x (2)	x		x	x	Rationally vs Empirically derived subscales
MFSI-SF	x	x (2)	x			x	
Myasthenia Gravis Fatigue Scale	x	x			x		
Neurological Fatigue Index for MS		x	x				Abnormal nocturnal sleep, relief by rest
Profile of Fatigue		x	x				
Revised Piper Fatigue Scale		x	x		x	x	
Schwartz Cancer Fatigue Scale		x	x			x	Temporal
Swedish Occupational Fatigue Inventory		x			x		Sleepiness, physical exertion, physical discomfort
Visual Analogue Scale for Fatigue^+^	x	x					
WEIMUS		x	x				
Wu Cancer Fatigue Scale		x	x			x	

*Synonymous names: Chalder Fatigue Scale, Fatigue Rating Scale, Fatigue Scale

^+^Synonymous name: Lee Fatigue Scale

### Assigning PROMIS fatigue items

All 95 items in the PROMIS fatigue item bank were reviewed by the primary author (KD), who then assigned them to one of the five dimensions. This classification was then reviewed by the second author (DLK) for consensus. If there was disagreement with any classification, a third reviewer (LS) was included to achieve consensus.

### Statistical analysis

The current PROMIS fatigue item bank was modeled to have one general fatigue factor with two local factors (experiences and impacts), which was psychometrically proven to be sufficiently unidimensional [10]. For this paper, in order to develop a content-specific CRF short form that produces scores comparable to the PROMIS fatigue item bank and its short forms, we first evaluated the sufficient dimensionality of the alternative model as discussed above using bi-factor analysis.

Bi-factor analysis includes two classes of factors: a general factor, defined by loadings from all of the items in the scale, and local factors, defined by loadings from pre-specified groups of items related to that sub-domain [22–25]. Items are considered sufficiently unidimensional when standardized loadings are > 0.3 for all the items on the general factor. Similarly, if the loadings of all the items on a local factor are salient, this would indicate that the local factor is well defined even in the presence of the general factor, and it is more appropriate to report scores of local factors separately [22, 24, 26].

In the model used by the current study, the general factor was “fatigue” and the 5 local factors were physical, cognitive, affective, global, and motivational. Once sufficient unidimensionality was supported, we then created a content-specific CRF short form by selecting items from each local factor by reviewing item content, as well as using item parameter threshold values obtained from item response theory (IRT) estimation, particularly the discrimination parameter.

Discrimination power describes the strength of an item’s discrimination between people at different fatigue levels below and above the threshold, indicating the degree of association between item responses and the fatigue latent trait. Items with the highest discrimination parameters typically produce the highest information function (i.e., lowest measurement errors) were considered the best candidates to be included in the short form.

## Results

The 95 items from the PROMIS fatigue bank were organized into the five fatigue dimensions (physical, cognitive, affective, global, and motivational) as listed in Table 2. After consensus was achieved, there were 12 items from the PROMIS fatigue bank that fit into the physical dimension, 13 in the cognitive dimension, 4 in the affective dimension, 32 for the global dimension, and 34 for the motivational dimension. Essential dimensionality of these items was supported with acceptable fit indices: RMSEA = 0.04, CFI = 0.985, TLI = 0.985. All items showed higher loading to the general factor than to their own local factor indicating the existing PROMIS item parameters are valid on this alternative model. See Table 3 for a summary of the PROMIS item selection information.

**Table 2:** Organization of Items from the PROMIS Fatigue Bank into Five Fatigue Dimensions

Physical	Cognitive	Affective	Global	Motivational
AN5	FATIMP02	AN15	AN1	AN3
FATEXP18	FATIMP06	FATEXP24	AN2	AN4
FATEXP19	FATIMP11	FATEXP26	AN8	AN7
FATEXP31	FATIMP14	FATEXP28	FATEXP02	AN12
FATEXP43	FATIMP17		FATEXP05	AN14
FATEXP44	FATIMP20		FATEXP06	AN16
FATEXP54	FATIMP22		FATEXP07	FATIMP01
FATIMP13	FATIMP30		FATEXP12	FATIMP03
FATIMP40	FATIMP35		FATEXP13	FATIMP04
FATIMP49	FATIMP38		FATEXP16	FATIMP05
FATIMP53	FATIMP43		FATEXP20	FATIMP08
HI12	FATIMP44		FATEXP21	FATIMP10
	FATIMP52		FATEXP22	FATIMP16
			FATEXP29	FATIMP15
			FATEXP34	FATIMP18
			FATEXP35	FATIMP19
			FATEXP36	FATIMP21
			FATEXP38	FATIMP24
			FATEXP40	FATIMP25
			FATEXP41	FATIMP26
			FATEXP42	FATIMP27
			FATEXP45	FATIMP28
			FATEXP46	FATIMP29
			FATEXP48	FATIMP34
			FATEXP49	FATIMP36
			FATEXP50	FATIMP37
			FATEXP51	FATIMP42
			FATEXP52	FATIMP45
			FATEXP56	FATIMP47
			FATIMP09	FATIMP48
			FATIMP33	FATIMP51
			HI7	FATIMP50
				FATIMP55
				FATIMP56

**Table 3:** PROMIS Item Selection Information

Item	Item Stem	Responses Scale*	Discrimination Parameter Value
Physical Domain			
FATIMP49	To what degree did your fatigue interfere with your physical functioning?	1	4.02
FATEXP43	How physically drained were you on average?	1	3.81
FATEXP19	How often were you physically drained?	2	3.65
FATIMP13	How often were you too tired to do errands?	2	3.51
FATEXP18	How often did you run out of energy?	2	3.39
AN5	I have energy	1	2.71
HI12	I feel weak all over	1	2.69
FATIMP53	How often were you too tired to take a short walk?	2	2.41
FATEXP54	How often did you have physical energy?	2	2.23
FATEXP31	How often were you energetic?	2	2.11
FATEXP44	How energetic were you on average?	1	1.98
FATIMP40	How often did you have enough energy to exercise strenuously?	2	1.17
Cognitive Dimension			
FATIMP20	How often did your fatigue make you feel less alert?	2	3.33
FATIMP17	How often did your fatigue make it difficult to make decisions?	2	3.26
FATIMP14	How often did your fatigue make it difficult to organize your thoughts when doing things at work (include work at home)?	2	3.17
FATIMP22	How often did your fatigue make it difficult to organize your thoughts when doing things at home?	2	3.13
FATIMP52	To what degree did your fatigue make you feel less alert?	1	3.11
FATIMP35	To what degree did your fatigue make it difficult to organize your thoughts when doing things at home?	1	3.09
FATIMP6	How often did your fatigue make you feel slowed down in your thinking?	2	2.97
FATIMP30	How often were you too tired to think clearly?	2	2.97
FATIMP43	To what degree did your fatigue make it difficult to organize your thoughts when doing things at work (include work at home)?	1	2.92
FATIMP2	To what degree did your fatigue make you feel slowed down in your thinking?	1	2.86
FATIMP38	To what degree did your fatigue make it difficult to make decisions?	1	2.81
FATIMP11	How often did your fatigue make you more forgetful?	2	2.71
FATIMP44	To what degree did your fatigue make you more forgetful?	1	2.36
Affective Dimension			
AN15	I am frustrated by being too tired to do the things I want to do	1	3.90
FATEXP26	How often were you too tired to enjoy life?	2	3.19
FATEXP28	How often were you too tired to feel happy?	2	3.04
FATEXP24	How often did you have enough energy to enjoy the things you do for fun?	2	2.11
Global Dimension			
FATEXP41	How run-down did you feel on average?	1	4.32
HI7	I feel fatigued	1	4.32
FATEXP35	How much were you bothered by your fatigue on average?	1	4.23
FATEXP40	How fatigued were you on average?	1	4.18
FATEXP22	How often were you bothered by your fatigue?	2	3.90
FATEXP34	How tired did you feel on average?	1	3.87
FATEXP36	How exhausted were you on average?	1	3.83
FATEXP51	How easily did you find yourself getting tired on average?	1	3.71
FATEXP56	What was the level of your fatigue on most days?	3	3.62
FATEXP48	How often did you find yourself getting tired easily?	2	3.51
FATIMP9	How often did your fatigue make it difficult to plan activities ahead of time?	2	3.48
FATEXP2	How often did you feel run-down?	2	3.42
FATEXP45	How sluggish were you on average?	1	3.39
FATEXP13	How bushed were you on average?	1	3.36
FATEXP52	How wiped out were you on average?	1	3.33
AN2	I feel tired	1	3.30
FATEXP7	How often did you feel your fatigue was beyond your control?	2	3.28
AN1	I feel listless (“washed out”)	1	3.27
FATEXP20	How often did you feel tired?	2	3.25
FATEXP29	How often did you feel totally drained?	2	3.09
FATIMP33	How often did your fatigue limit you at work (include work at home)?	2	3.09
FATEXP12	To what degree did you feel tired even when you hadn’t done anything?	1	2.96
FATEXP38	How fatigued were you on the day you felt most fatigued?	1	2.92
FATEXP6	How often did you feel tired even when you hadn’t done anything?	2	2.84
FATEXP21	How fatigued were you when your fatigue was at its worst?	1	2.83
FATEXP49	How often did you think about your fatigue?	2	2.73
FATEXP5	How often did you experience extreme exhaustion?	2	2.66
FATEXP16	How often were you sluggish?	2	2.65
FATEXP50	How fatigued were you on the day you felt least fatigued?	1	1.91
AN8	I need to sleep during the day	1	1.64
FATEXP46	On how many days was your fatigue worse in the morning?	4	1.49
FATEXP42	How much mental energy did you have on average?	1	1.44
Motivational Dimension			
FATIMP3	How often did you have to push yourself to get things done because of your fatigue?	2	4.77
AN3	I have trouble <U>starting</U> things because I am tired	1	4.35
FATIMP1	To what degree did you have to push yourself to get things done because of your fatigue?	1	4.08
FATIMP50	Did fatigue make you less effective at home?	1	4.00
FATIMP16	How often did you have trouble finishing things because of your fatigue?	2	3.86
FATIMP27	To what degree did you have trouble starting things because of your fatigue?	1	3.82
FATIMP24	How often did you have trouble starting things because of your fatigue?	2	3.81
FATIMP48	To what degree did your fatigue interfere with your social activities?	1	3.81
FATIMP51	To what degree did you have trouble finishing things because of your fatigue?	1	3.80
FATIMP37	Due to your fatigue were you less effective at work (include work at home)?	1	3.79
FATIMP4	How often did your fatigue interfere with your social activities?	2	3.71
FATIMP10	How often did your fatigue make it difficult to start anything new?	2	3.71
FATIMP47	To what degree did you have to force yourself to get up and do things because of your fatigue?	1	3.68
FATIMP36	To what degree did your fatigue make it difficult to start anything new?	1	3.68
AN16	I have to limit my social activity because I am tired	1	3.61
FATIMP15	How often did your fatigue interfere with your ability to engage in recreational activities?	2	3.56
FATIMP18	How often did you have to limit your social activities because of your fatigue?	2	3.53
FATIMP42	How often were you less effective at home due to your fatigue?	2	3.52
FATIMP5	How often were you less effective at work due to your fatigue (include work at home)?	2	3.52
FATIMP55	How often did you have to force yourself to get up and do things because of your fatigue?	2	3.51
FATIMP19	How often were you too tired to do your household chores?	2	3.41
AN4	I have trouble <U>finishing</U> things because I am tired	1	3.40
FATIMP34	To what degree did you have to limit your social activities because of your fatigue?	1	3.29
FATIMP45	To what degree did your fatigue interfere with your ability to engage in recreational activities?	1	3.24
FATIMP26	How often were you too tired to socialize with your family?	2	3.11
FATIMP29	How often were you too tired to leave the house?	2	3.09
FATIMP56	How often were you too tired to socialize with your friends?	2	2.87
FATIMP25	How often was it an effort to carry on a conversation because of your fatigue?	2	2.84
FATIMP28	How hard was it for you to carry on a conversation because of your fatigue?	1	2.81
AN7	I am able to do my usual activities	1	2.55
AN12	I am too tired to eat	1	2.31
AN14	I need help doing my usual activities	1	2.31
FATIMP21	How often were you too tired to take a bath or shower?	2	2.11
FATIMP8	How often were you too tired to watch television?	2	1.70

*Response scale 1: 1, not at all; 2, a little bit; 3, somewhat; 4, quite a bit; 5, very much

Response scale 2: 1, never; 2, rarely; 3, sometimes; 4, often; 5, always

Response scale 3: 1, none; 2, mild; 3, moderate; 4, severe; 5, very severe

Response scale 4: 1, none; 2, 1 day; 3, 2–3 days; 4, 4–5 days; 5, 6–7 days

**Fig. 1:**
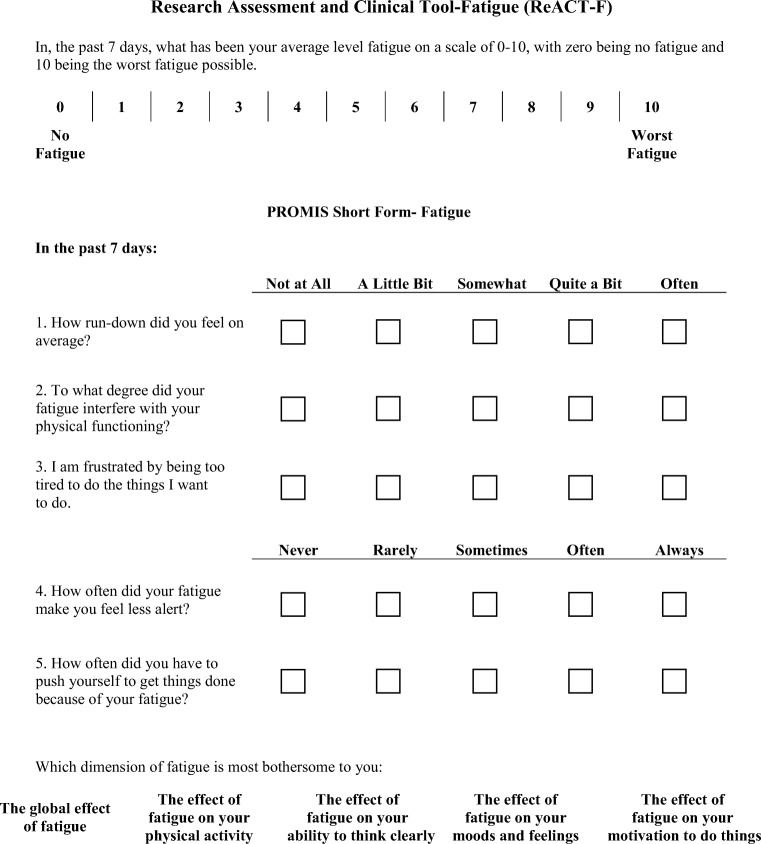
Research Assessment and Clinical Tool-Fatigue (ReACT-F). To obtain permission to use, please contact the corresponding author© K. Dickinson, D. Lynch Kelly, J-S. Lai, L. Saligan

The PROMIS-based Research Assessment and Clinical Tool-Fatigue (ReACT-F) CRF-specific short form was created using established item parameters (Fig. 1). Each item was selected based upon the discriminative value and is considered representative to each fatigue dimension. We added a numeric rating scale at the beginning of the questionnaire per the current fatigue assessment guidelines and we added an additional item, “*Which aspect of fatigue is most bothersome to you*” to assess the overall burden of the CRF dimensions and inform treatment decisions to optimize CRF management.

### Scoring

This short form can be scored using similar approaches as used by other PROMIS fatigue short forms [27]. A 5-item questionnaire can only be scored when at least 4 of the items are completed. However, patients should be encouraged to complete all items to minimize measurement errors. Each question has a Likert scale with values ranging from one to five. A total raw score is calculated by summing the five items on the questionnaire and a prorated value will be used to replace missing value; therefore, the total score on the instrument ranges from 5 to 25. Higher scores indicate worse fatigue. A raw score can then be translated into a PROMIS based *T*-score to create the final score for a respondent (details are shown in www.healthmeasures.net/score-and-interpret/calculate-scores).

## Discussion

The purpose of this paper was to develop a brief tool to capture the multidimensional nature of CRF. This was carried out by examining items included in the 95-item PROMIS fatigue bank to determine if specific items could be selected to measure different fatigue dimensions. The final CRF assessment tool, the ReACT-F, consists of five PROMIS items, where each item is expected to screen a specific dimension of CRF: physical, cognitive, affective, global, and motivational.

The ReACT-F is a self-report short form that addresses the previously identified gap in the literature, which is the lack of a brief, clinically useful tool to quickly assess the multidimensional nature of fatigue in the cancer population. This new content-specific short form requires further validation to determine its clinical and scientific relevance. In the clinic, it is expected that the ReACT-F can aid clinicians to quickly assess the specific fatigue experience of their patients to allow for a more focused evaluation and tailored management. For example, patients reporting physical fatigue may be further evaluated for deconditioning, cardiopulmonary status, or musculoskeletal impairment, so physical rehabilitative strategies can be planned. Individuals who report affective fatigue may be referred for comprehensive psychological evaluation, while those who report affective fatigue or cognitive fatigue may benefit from occupational psychotherapy for behavioral adaptive coaching and a neuropsychology consult for comprehensive cognitive function evaluation, respectively.

Scientifically, this evaluation tool will be useful to determine the phenotypic characteristics of each fatigue dimension within the global fatigue construct. The ReACT-F tool can assist in identifying clinical and demographic attributes, as well as the biologic profile of the specific fatigue experience, to advance our understanding of the etiology of CRF. Understanding the etiology of CRF is important for treatment development and generation of algorithms to identify individuals at risk to develop clinically meaningful fatigue related to the progression of their disease or as a side effect of their treatment.

### Limitations

The five items have high discriminative value demonstrating the ability to allow for the assessment of multidimensions of fatigue; however, they did not have sufficient power when factor loading to be independent from the construct of fatigue, as assessed through comparison of factor loadings between the general factor (fatigue) and the local (subdomain) factors. Thus, the dimensions are not independent constructs, but components of a general fatigue construct.

## Conclusions

In conclusion, the ReACT-F is a CRF-specific self-report short form that addresses the need for a brief, clinically useful tool to quickly assess the multidimensional nature of CRF. The ReACT-F assesses five common dimensions of CRF as well as perceived burden of the fatigue dimensions. This tool has clinical and scientific promise, to advance our understanding and management of CRF. We anticipate that the ReACT-F can be completed in the clinical setting in approximately 3 minutes, providing clinicians with meaningful data to drive personalized interventions. Further validation of the ReACT-F is highly encouraged to assess its psychometric properties and determine its clinical utility.
